# Evaluation of Iron deficiency anemia and BMI in children suffering from Helicobacter pylori infection

**Published:** 2014-12-10

**Authors:** H Bazmamoun, Z Razavi, H Esfahani, Arefian

**Affiliations:** 1 Associate professor of pediatric gastroenterology, Department of pediatrics, Hamedan University of Medical Sciences, Hamedan, Iran.; 2 Associate professor of pediatric endocrinology, Department of pediatrics, Hamedan University of Medical Sciences, Hamedan, Iran.; 3 Assistant professor of pediatric hematology/oncology, Department of pediatrics, Hamedan University of Medical Sciences, Hamedan, Iran.; 4 General physician, Hamedan University of Medical Sciences, Hamedan, Iran.

**Keywords:** Helicobacter pylori, iron deficiency anemia, body mass index, children

## Abstract

**Background:**

Recent studies suggest an association between H. pylori infection and disorders such as iron deficiency anemia and growth delay. Considering the high prevalence of H. pylori infection and iron deficiency anemia, this study was performed in order to evaluate their relevance in children undergoing an upper endoscopy.

**Materials and Methods:**

In this case-control study, children aged 2 to 16 years old, undergoing endoscopy from March 2012 to March 2013 at Besat Hospital of Hamedan, were selected. Participants were divided in H.Pylori infected and non-infected groups. Then the two groups were compared in terms of body mass index (BMI) and the incidence of iron deficiency anemia. The presence of Helicobacter pylori infection in children was confirmed by Giemsa staining of gastric biopsy specimens. Collected data was analyzed by SPSS 17.0 (SPSS Inc., Chicago, IL) and t-test and chi-square.

**Results:**

In this study, 200 children (94 male and 106 female) were evaluated. The most common presenting symptom in both groups was abdominal pain. 8.2 % (9 cases) of the infected patients and 10.5% (10 cases) of the non-infected patients had iron deficiency anemia which this difference was not statistically significant (p=270). Also, no statistically significant difference was noted between the two groups in terms of gender (p=0.32), hemoglobin (p=0.35), Ferritin levels (p= 0.275) and body mass index (p= 0.273).

**Conclusion:**

The results of this study not showed an association between H. pylori infection and iron deficiency anemia or body mass index in studied children

## Introduction

Iron deficiency is the most common and widespread nutritional disorder in the world. It is estimated that 30% of the global population suffers from iron-deficiency anemia, and most of them live in developing countries (1). Iron deficiency anemia can lead to dysfunction in immune, cognitive and motor systems (1, 2, 3, 4). 

H. pylori is one of the most common bacterial infections in developing countries which usually affects children and if not treated usually continues to exists in the body for a long time(5). This bacterium can cause gastrointestinal disorders such as chronic active gastritis, stomach cancer, peptic ulcer and Malt lymphoma. It is classified in group 1 carcinogens by WHO (1, 6, 7). Short stature, sudden infant death sundrome and ITP have been reported as its extraintestinal manifestations(1,8,9).

In some studies association between H. pylori infection and iron deficiency anemia has been described and successful treatment of H.Pylori has led to improvement of anemia and serum Iron. Also in some studies, H.pylori has been suggested as the cause of refractory cases of IDA which improved with eradication of H. pylori infection (10-17).

There is still disagreement about the association between H. pylori infection and iron deficiency anemia and some studies have not shown this association (18-20). In a study was conducted in Iran by Hoseinzadeh et al, with using anti H.pylori antibodies in 2010, this association was shown (21), but in another study that conducted in Iran by Zamani A et al, in 2011, no association was detected (22).

In this study we tried to assess the association between H. pylori infection and Iron deficiency anemia in Iranian infected children by direct study of H.pylori in tissue samples

## Material and methods

In this case control study, the statistical population included all 2-16 year old children (102 H Pylori infected children and 98 non-infected ones), undergoing upper endoscopy by recommendation of pediatric Gastroenterologist. These children were admitted with chronic abdominal pain, nausea and vomiting to Besat hospital of Hamadan, Iran, between March 2012 and March 2013.

Patients with upper and lower gastrointestinal bleeding and underlying blood disorders , female adolescents with severe menstrual bleeding, patients treated with anti H.pylori medications or Iron supplements and patients whose parents did not have adequate cooperation in giving history were excluded from the study.

The study was started after necessary explanation regarding the project to the parents and obtaining consent forms. Endoscopy and biopsy were performed by a pediatric gastroenterologist and obtained samples were assessed by a pathologist. Geimsa staining was used to assess H.Pylori infection in the obtained samples and updated Sydney histological scoring system (23) was used for determining the grading of gastritis.

Simultaneously, IV blood sample was obtained from basilic vein to measure serum Ferritin and CBC. Serum Ferritin was measured by ELISA method (Mono bind company kit made in USA was used) and CBC test was performed by Sysmex cell counter kx-21 made in Japan. 

Iron-deficiency anemia was diagnosed with serum Ferritin less than 12 ng/ml and Hb below normal range (for the age and gender)(24), The children height and weight were measured by one person. Height was measured in standing position without shoes by using a metal meter stick while heels of the feet were put together and against the wall then recorded in the questionnaire Based on the results of the biopsy specimens, patients were divided in H.Pylori positive and H .Pylori negative group. 

**Statistical Analysis**

The collected data was analyzed by using SPSS 17.0 (SPSS Inc., Chicago, IL) and descriptive statistics and frequency distribution tables. Patents were compared using t-test and chi-square. P<0.05 was considered statistically meaningful.

## Results

This study was performed on 200 children, aged 2-16 years old admitted to Besat hospital of Hamadan, Iran, between March 2012 and March 2013.Their presenting symptoms were chronic abdominal pain, chronic nausea and vomiting. The need for endoscopy was determined by a pediatric Gastroenterologist.

The mean age of the participants was 9.3 + 3.41 years .Among 200 patients, 94 patients (% 47) were male and 106 patients (% 53) were female. The most common presenting symptom in both groups was abdominal pain (87%).

Among infected patients 8.2 % (9 cases) and among non-infected patients 10.5 % (10 cases) were suffering from Iron deficiency anemia.

As shown in table 1, in terms of hemoglobin level (12/76±1/00 g/dl in infected patients and 9/60±1/53 g/dl in non-infected patients), serum 

Ferritin (36/00±19/54 ng/ml in infected patients and 41/52±22/68 ng/ml in non-infected patients) and BMI (16/62±2/19 in infected patients and 16/05±1/06 in non-infected patients), there was no meaningful difference between 2 groups.

**Table I:** Comparison of the age, hemoglobin level, serum ferritin and BMI between the parcipitants.

	**H-pylori(+)**	**H-pylori(-)**	**P Value**
**Age**	9/05±3/19	9/60±1/53	0/271
**Hemoglobin** **(g/dl)**	12/76±1/00	13/01±1/18	0.35
**Serum ferritin** **(ng/ml)**	36/00±19/54	41/52±22/68	0.275
**BMI**	16/62±2/19	16/05±1/06	0.273

## Discussion

According to this study results, there is no effect of gender on the prevalence of H.Pylori infection which is similar to the results of Keramati et al’ study in Mashad(25), but in some studies the incidence of H.Pylori infection in the boys was significantly higher than the girls(20,22). 

With respect to correlation between H.Pylori infection and BMI, our data failed to show the significant difference between 2 groups which is similar to the study of Ozlem Bekem et al, based on endoscopic finding in children with dyspepsia (26). But In another study conducted in Mexico, H pylori infection was associated with growth delay in children (8).

The present study not has shown a meaningful difference in incidence of IDA between the groups. Also no meaningful difference was noted on variables such as average level of serum Ferritin and Hb in children of 2 groups. In Seo et al’ study in Korea, conducted on 753 children aged 6-12 year old in 2002, serum Ferritin level was lower and the incidence of IDA was higher in H.Pylori -positive serology children(27).

In one study conducted by Fakheri and associates in Sary city in Iran on 400 children using serological methods, serum Ferritin and Iron in H.Pylori infected children were lower than the control group (28). Two other studies, one in Denmark and another one in the USA, showed that rate of IDA in H.Pylori infected people was significantly lower than non-infected group (29, 30).

In some other studies including the present one, no significant correlation was detected between IDA and H.Pylori infection. In Collet and associates’ study conducted on adult participants in Australia, no significant difference was noted between serum Ferritin levels of H.Pylori infected men and women and non-infected ones(30). In another study performed by Sarker and associates in Bangladesh, there was no correlation between IDA and H.Pylori infection (32).

Also in one study conducted in Imam Reza Hospital of Mashad on 184 patient, no significant difference was detected between average serum Ferritin level of H.Pylori infected group and control group(26).

## Conclusion

Overall, in spite of conducted studies, the role of H.pylori in developing IDA and possible effective mechanisms, involved, is still a challenging issue. It seems in studies such as the present one which both case and control group participants were selected from symptomatic children ,the role of other possible causes of appetite loss in developing IDA and growth delay should be taken into consideration. Also factors such as duration of suffering from gastritis, Helicobacter pylori strain type and dietary status of participants in the study should be considered in interpretation of study results. 

Conducting more studies, performing clinical trials and cohort studies, assessment of anemia before and after treatment of H.Pylori infection are among strategies which can lead to further clarification of different aspects of this issue.
